# Bowel obstruction due to *Chlamydia trachomatis*: a case report and review of literature

**DOI:** 10.1186/s40792-021-01130-w

**Published:** 2021-02-15

**Authors:** Yuta Shibasaki, Makoto Sohda, Hiroomi Ogawa, Chika Katayama, Naoya Ozawa, Chika Komine, Kunihiko Suga, Katsuya Osone, Takuhisa Okada, Takuya Shiraishi, Ryuji Katoh, Takehiko Yokobori, Akihiko Sano, Makoto Sakai, Ken Shirabe, Hiroshi Saeki

**Affiliations:** 1 Department of General Surgical Science, Division of Hepatobiliary and Pancreatic Surgery, Graduate School of Medicine, Gunma University, 3-39-22 Showa-Machi, Maebashi, Gunma 371-8511 Japan; 2 Innovative Medical Research Center, Gunma University Hospital, Graduate School of Medicine, Gunma University, 3-39-22 Showa-Machi, Maebashi, Gunma 371-8511 Japan

**Keywords:** *Chlamydia trachomatis*, Pelvic inflammatory disease, Bowel obstruction

## Abstract

**Background:**

Chlamydial infection is a difficult-to-diagnose type of sexually transmitted disease that occurs mainly in young people. We report a case of bowel obstruction caused by intrapelvic adhesions formed by chlamydial infection.

**Case presentation:**

This patient was a 23-year-old woman who had been suffering from acute abdominal pain. She had been previously treated several times for intrapelvic abscesses and had a history of chlamydial infection. Endometriosis was thought to be the cause of her pelvic abscess based on endoscopic findings. Computed tomography demonstrated a small bowel obstruction caused by a pelvic abscess. However, the diagnosis could not be confirmed. She underwent laparoscopic surgery and was diagnosed with bowel obstruction due to adhesion of chlamydial infection based on the intraoperative findings and *Chlamydia trachomatis* antibody test. She was discharged 5 days after surgery.

**Conclusions:**

It is necessary to consider the possibility of chlamydial infection as a cause for lower abdominal pain and unexplained bowel obstruction in female patients.

## Background

Chlamydial infection is a type of sexually transmitted disease (STD) that occurs mainly in young people, and its diagnosis is often difficult due to high frequency of asymptomatic infections [1]. Chlamydial infection can cause pelvic infection disease (PID), ovarian tube abscess, and involves liver capsule inflammation associated with PID [2]. These have been reported as possible causes of adhesion, bowel obstruction, and infertility [2–4]. Although there are few reports of laparoscopic surgery, laparoscopic surgery may be useful in its diagnosis and may make treatment less invasive [1, 2]. We present a patient who successfully underwent laparoscopic surgery for adherent bowel obstruction due to chlamydial infection.

## Case presentation

This patient was a 23-year-old woman who had acute abdominal pain. She had been treated for intrapelvic abscesses several times previously at another hospital. She also had a history of STD due to chlamydial infection. Computed tomography (CT)-guided drainage was performed, and the patient's symptoms improved. Bloody purulent fluid was noted, and *Escherichia coli*, but not *Chlamydia*, was detected by culture test. Polymerase chain reaction (PCR) using urine sample was negative for chlamydial infection when the patient was being treated for pelvic abscesses. In addition, a lower gastrointestinal endoscopy was performed to closely examine the source of infection, and intestinal findings suspected endometriosis. Endometriosis is a condition in which endometrium occurs in areas other than the uterus, such as the Douglas fossa, ovaries, intestinal tract, and pelvis. These areas may become infected, resulting in abscess formation. She also had recurrent pelvic abscess, which suggested endometriosis as the cause of the pelvic abscess [5, 6]. While the patient was being medically treated for endometriosis, she had repeated pelvic abscesses. Thus, she was scheduled to be referred to the obstetrics and gynecology department in our institute for further investigation and treatment.

**Fig. 1:**
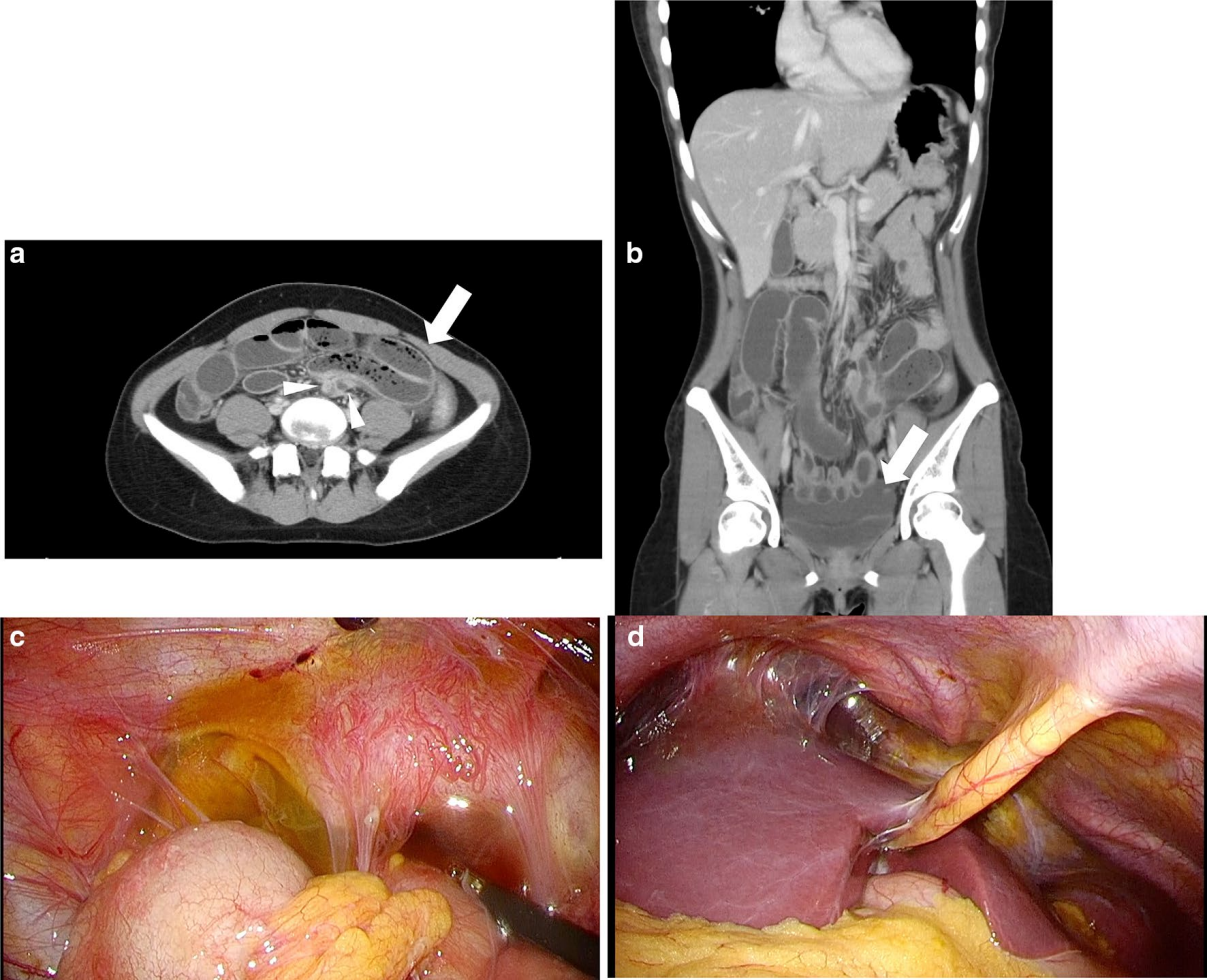
Abdominal CT and intraoperative findings. **a** arrow: Closed loop formed and dilated small intestine, arrowheads: Collapsed small intestine on the oral and anal sides of the closed loop, **b** ascites in the pelvis, **c** ascites in the pelvis and adhesion of the uterine appendages, and **d** adhesions that are translucent and considered to be in the active stage of chlamydial infection on the liver surface

Meanwhile, she had a relapse of abdominal pain and was taken to our hospital in an emergency. Palpation of the abdomen revealed localized tenderness below the umbilicus and peritoneal irritation symptoms. CT demonstrated a small bowel obstruction with closed loop (Fig. 1a) and a small amount of intrapelvic abscess (Fig. 1b). Contrast enhancement of the liver surface was also recognized. Laboratory results included white blood cell and platelet counts of 12,800/μL and 224,000/μL, respectively. The prothrombin time and international normalized ratio was 0.97. Total serum bilirubin, albumin, aspartate aminotransferase, alanine aminotransferase, alkaline phosphatase, and C-reactive protein was 1.6 mg/dL, 4.3 g/dL, 22, 35, 239 U/L, and 0.71 mg/dL, respectively (Table. 1).

**Table 1.** Laboratory results

Blood count		Blood chemistry	
WBC	12,800/μL	T-bil	1.6 mg/dL
Plt	224,000/μL	Alb	4.3 g/dL
		AST	22 U/L
		ALT	35 U/L
**Blood coagulation system**		ALP	239 U/L
PT-INR	0.97	CRP	0.71 mg/dL

Taking into consideration the patient’s history and clinical findings, the patient was suspected to have bowel obstruction due to pelvic abscesses caused endometriosis infection by *Escherichia coli*, but the diagnosis could not be preoperatively confirmed.

Laparoscopic surgery was performed for diagnostic and therapeutic purposes. Fibrous adhesions from the liver to the pelvis was intraoperatively recognized. Adhesions in the lower part of the umbilicus formed a small bowel loop, and fibrous adhesions and ascites were also found in the pelvis (Fig. 1c, d). The uterus could be confirmed, but not the ovaries due to adhesions in the pelvis. Adhesions causing the small bowel loop were found in the small bowel 100 cm from the Treitz ligament. In addition, multiple other adhesions and bands were found throughout the abdominal cavity. The band due to the adhesions was detached and the bowel obstruction was released. The entire small bowel was then checked and confirmed that there were no other abnormalities. The small bowel was edematous, but not necrotic, and bowel resection was not performed. Intraoperatively, ascitic fluid in the abdominal cavity, including in the pelvis, was aspirated and cleaned as much as possible. There was no obvious abscess formation.

*Chlamydia trachomatis* antibody IgA was found to be positive, and IgG was negative as shown in the blood sample preoperatively submitted. Based on the typical intraoperative findings and the results of *C. trachomatis* antibody, she was finally diagnosed with small bowel obstruction caused by the adhesion of chlamydial infection. As for the antibiotics used for treatment, Levofloxacin was used for chlamydial infection. In the perioperative period, Cefmetazole and Clindamycin Phosphate were used for anaerobic infections such as *Escherichia coli*. Her symptoms improved by adhesion detachment. The postoperative course was uneventful, and the patient was discharged 5 days after surgery.

## Discussion

The strains of *C. trachomatis* are divided into three biovars and are further subtyped by serovars. Of those, the genital tract biovar (serovars D–K) is associated with the most prevalent STD [7]. In women, 70–80% of genital tract infections with *C. trachomatis* are asymptomatic, but 15–40% ascend to the upper genital tract, which can lead to serious sequelae, including PID, tubo-ovarian abscess, perihepatitis (Fitz-Hugh–Curtis syndrome, FHC), infertility, and ectopic pregnancy [8, 9].

Also, it has been reported that genital tract infections with *C. trachomatis* can cause adhesions and bowel obstruction [1, 9, 10]. Contrast-enhanced CT shows a characteristically dense subhepatic staining in the early phase. A fibrous adhesion, known as violin string adhesion, is a typical intra-abdominal finding of FHCS [2]. And the positive rate of chlamydial intra-abdominal infection in the presence of violin string adhesion has been reported to be more than 80% [11]. In our case, we also observed a contrast enhancement of the liver surface on CT and characteristic intra-abdominal findings such as violin string adhesion on the liver. A slightly purulent ascites in the pelvis was also observed, although the cause was not clear at the point of operation. In this case, IgA was positive and IgG was negative, so the antibody test indicates that the patient was in the active stage of chlamydial infection, which may be early enough for fibrotic adhesions to occur. The intraoperative findings of translucent membranous tissue of perihepatic violin string adhesion and other intra-abdominal adhesions suggest that the patient was still in the active stage. On the other hand, it is also suggested that bowel obstruction due to chlamydial infection can occur even in the active stage, as in this case. Although the possibility that the IgG was false negative and the time of the negative IgG coincided with the early stage of IgA-positive infection were also considered, we comprehensively judged that the bowel obstruction, including the intraoperative findings, was caused by active chlamydial infection [7].

Adhesions (74%), Crohn’s disease (7%), neoplasia (5%), hernia (2%), radiation (1%), and miscellaneous (11%), are the etiology of bowel obstruction [12]. Among them, bowel obstruction due to chlamydial infection is extremely rare, and preoperative diagnosis is considered difficult. Bowel obstruction due to chlamydial infection was first described in 1899, and, so far, only seven cases including our case have been reported in the English literature (Table. 2). Including our case, surgery was performed in five cases for diagnostic and therapeutic purposes, suggesting the difficulty of preoperative diagnosis. On the other hand, two cases improved with conservative treatment with antibiotics and drainage, including a gastric tube [13, 14]. Only one case obtained preoperative diagnosis as bowel obstruction due to chlamydial infection because the CT showed findings of suspected FHC on the liver surface [3].

**Table 2:** Previous reports of bowel obstruction due to chlamydial infection

No	Year	Author	Age	Sex	Chief complaint	History of laparotomy	Chlamydia antibody etc	CT or X-ray	Preoperative diagnosis	Treatments
1	1990	McCormick M	26	Female	Abdominal pain	ND	Positive	Colonic Obstruction		Antibiotics Anorectal drain
2	1990	Pegg DJ	18	Female	Abdominal pain Vomiting	ND	ND	Bowel Obstruction	Bowel Obstruction	Bowel obstruction release
3	2003	Harel Z	19	Female	Abdominal pain Vomiting	None	Vaginal Culture Positive	Small bowel Obstruction		Antibiotics Gastric tube
4	2006	Oh SN	60	Female	Abdominal pain Vomiting	None	Positive	Small bowel Obstruction FHC Chilaiditi syndrome	Small bowel Obstruction FHC Chilaiditi syndrome	Small bowel resection
5	2015	Cusimano A	40	Female	Abdominal pain	None	PCR Positive	Bowel Obstruction Intestinal Mal-rotation	Bowel Obstruction Intestinal Mal-rotation	Laparoscopy Bowel obstruction release
6	2019	Haumann A	27	Female	Abdominal pain Vomiting	None	PCR Positive	Small bowel Obstruction	Small bowel Obstruction	Laparoscopy
7	2019	Our case	24	Female	Abdominal pain	None	Positive	Small bowel Obstruction	Small bowel Obstruction	Laparoscopy Bowel obstruction release

*ND* no date, *FHC* Fitz-Hugh–Curtis Syndrome, *PCR* Polymerase chain reaction

As shown in Table 1, laparoscopic surgery has been performed in three cases with bowel obstruction due to chlamydial infection. In one case of the two open surgery, small bowel resection was performed [3, 4]. However, in laparoscopic cases, only a band resection was performed to release the bowel obstruction, and no small bowel resection was performed. Recently, laparoscopic surgery tends to be performed for patients with intestinal obstruction resistant to conservative treatment, especially for with unknown cause [1]. In our case, fibrous cordage could be dissected by laparoscopic surgery, and the bowel obstruction was resolved. Furthermore, the findings of PID were confirmed in the pelvis and on the liver surface. The advantages of choosing laparoscopic surgery may not only be because of its less invasiveness but also because of its suitability for adequate intraperitoneal observation. The methods of testing for *C. trachomatis* include cell culture methods using cervical abrasion or urine samples from the affected area, antigen tests (e.g., enzyme immunoassay), genetic tests (e.g., PCR), and serum antibody tests. Cell culture is reported as the standard for testing, but the condition of the facility, the skill, and the cost should be considered. A combination of antigen and genetic tests are generally used to diagnose the current infection [15]. Serum antibody testing should be used as an adjunct because antibodies may not be elevated early in the course of infection [16]. However, serum antibody testing may be useful in cases where specimen collection is difficult (e.g., pelvic infections and pregnant women) and for infertility screening [17]. In our case, neither cell culture from the abscess nor PCR-detected *C. trachomatis* because timing of the chlamydial infection was subclinical. With regard to adhesive bowel obstruction caused by chlamydial infection, a history of infection proven by serum antibody testing, as well as the current infection, may be useful for diagnosis. Both these intraoperative findings and the positivity of *C. trachomatis* antibody IgA, that was proved postoperatively, led to the diagnosis of bowel obstruction due to chlamydial infection. Testing should be considered to differentiate chlamydial infection for cases with bowel obstructions with unknown cause in women. Chlamydial infection may be suspected, especially in young women, as in this case, or if CT findings indicate that the site of obstruction is slightly above the pelvis.

## Conclusions

We performed laparoscopic surgery for bowel obstruction due to chlamydial infection. Chlamydial infection must be considered for patients with bowel obstruction of undetermined origin.

## Data Availability

All data generated or analyzed during this study are included in this published article.

## Abbreviations

STD: Sexually transmitted disease
CT: Computed tomography
PID: Pelvic infection disease
FHC: Fitz-Hugh–Curtis syndrome
